# Piikun: an information theoretic toolkit for analysis and visualization of species delimitation metric space

**DOI:** 10.1186/s12859-024-05997-y

**Published:** 2024-12-18

**Authors:** Jeet Sukumaran, Marina Meila

**Affiliations:** 1 Biology, San Diego State University, San Diego, CA USA; 2 Statistics, University of Washington, Seattle, 10587 WA USA

**Keywords:** Evolutionary biology, Species delimitation, Information theory, Metrics, Distances

## Abstract

****Background**:**

Existing software for comparison of species delimitation models do not provide a (true) metric or distance functions between species delimitation models, nor a way to compare these models in terms of relative clustering differences along a lattice of partitions.

****Results**:**

Piikun is a Python package for analyzing and visualizing species delimitation models in an information theoretic framework that, in addition to classic measures of information such as the entropy and mutual information [1], provides for the calculation of the Variation of Information (VI) criterion [2], a true metric or distance function for species delimitation models that is aligned with the lattice of partitions.

****Conclusions**:**

Piikun is available under the MIT license from its public repository ( https://github.com/jeetsukumaran/piikun), and can be installed locally using the Python package manager ‘pip‘.

## Background

The field of species delimitation – computational approaches to determining the fundamental units of nature and atomic units of analysis in fields such as evolutionary biology and phylogenetics – has rapidly advanced in a number of ways recently, including machine learning approaches [3], incorporation of natural history [4], hierarchically modeling the speciation process over a primary population-level structuring [5] as well as other biological criteria [6].

Species delimitation inferential analysis results in a particular organization or (set theoretic) partition of a set of samples of a biological system into (mutually-exclusive and jointly-comprehensive) subsets, with each subset corresponding to a species unit, nominal or otherwise [7]. It is customary to reference each distinct partition of the system as a “species delimitation model”, and species delimitation analysis can be seen as a model selection procedure under some optimality criterion given a particular dataset. Despite advances in the field of species delimitation inference each novel method and software package, we still lack a true metric space to compare and relate the results of these various species delimitation models to each other in terms of their similarities or differences. While indexes such as the taxonomic index of congruence ($C_{tax}$) [8] or the match ratio [9], implemented in [10], are useful, they do not satisfy all properties of a metric, such as the triangle inequality [11]. Other indexes are restricted to cases involving nested models [8].

The absence of true distance characteristics, like the triangle inequality, complicates intuitive interpretation when comparing more than two species delimitation models. Beyond human interpretation, metricity can be useful when used as a summary statistic in machine learning. Many machine learning algorithms, especially those involving clustering, classification, or dimensionality reduction, rely on the triangle inequality and other characteristics resulting from well-defiend distance functions to effectively optimize solutions. As with human interpretation, when distance measures violate properties like the triangle inequality, comparisons among three or more species delimitation models can become inconsistent or ambiguous. A true metric space allows for better generalization in machine learning by providing meaningful and consistent distances between unseen species delimitation models. Without such a framework, models trained on species delimitation data may fail to generalize well, as the inconsistencies in distance measures affect the algorithm’s ability to correctly interpret new models or datasets. A true metric between species delimitation models will also support the design and fine-tuning of proposals for moving through MCMC space – e.g., by allowing for proposal of candidate partitions that are more similar to the current when sampling a better part of parameter space.

In contrast to the limited number of indexes of comparison available for species delimitation models, let alone lack of a metric, the related field of phylogenetics has had metrics for evolutionary trees since at least 1981 [12], and since then there has been continuous growth in diversity, including extensions that allow for comparisons between trees that only share a subset of taxa [13, 14], multi-labeled trees or trees with multiple occurences of the same label [15], or fully-labeled trees (tree with internal nodes labeled) with potentially no overlapping leaves at all [16, 17]. Some especially remarkable advances gained in quantifying distances between evolutionary trees using information theoretic or (Shannon) entropy-based approaches [1]: the classical Robinson-Foulds (RF) distances [12] have been extended using information measures developed by Steel and Penny [18] as well as the variation of information (VI) clustering criteria of Meila [2], producing information theoretic tree RF distances by Smith [19]. Information geometric approaches have also been used to develop metrics for comparing distances between trees in genetic sequence probability space or ”wald space” [20, 21], coalescent or gene tree probability space [22], and continuous trait evolution model probability space [23].

The potential for insight gained by these advances in phylogenetic applications cannot be underestimated, both in the theoretical as well as empirical context. Here we adopt the same information theoretic approaches that have proven successful in discriminating between evolutionary trees (in particular, [2]; see [24] for a review) as the basis for providing the first metric space for species delimitation models in the software package reported here, Piikun.

## Implementation

This paper describes Piikun^1^, a pure Python package [26] for the analysis and visualization of species delimitation models in an information theoretic framework that provides a true distance or metric space for these models. The package is publically available for download or local installation using ‘pip‘ from its GitHub website https://github.com/jeetsukumaran/piikun, and depends on the following libraries: NumPy [27], SciPy [28], PANDAS [29, 30], plotly [31], Matplotlib [32], Seaborn [33].

The models analyzed using Piikun may be generated by any inference package, such as BPP [34], DELINEATE [5] etc., or taxonomies or classifications based on conceptual descriptions in literature, geography, speculation, etc. Regardless of source or basis, each of these ways to organize or cluster a set of lineages into a set of higher-level units is a (set theoretic) *partition* of those lineages [5, 7], and can be described in numerous ways that Piikun can read (e.g., a generic JSON dictionary, or the a species delimitation model data exchange format “SPART-XML” [7]). Piikun further supports specialized input formats, such as the comprehensive results file from DELINEATE [5] or BPP [34] analyses, which allow for incorporation of additional information, such as support values, as shown below.

Piikun provides a range of univariate information theoretical statistics for each individual model in the input set (e.g., the entropy [1]), as well as bivariate statistics (e.g., the mutual information, joint entropy, [1]) for each distinct pair of these models, as well as *true* metrics (distances) between every pair of species delimitation models based on these information theoretic measures: the variation of information [2] and the normalized joint variation of information distance [35].

### The variation of information partition distance

Every species delimitation model is a *partition* of a set of lineages into a set of mutually exclusive and jointly comprehensive subsets [7]. As such, the *variation of information* criterion of [2], which provides a true distance function for partitions, also establishes a metric space for species delimitation models. Given two partitions, $\psi ^u, \psi ^v$, $VI(\psi ^u, \psi ^v)$, this is defined as [2]:

1 $$\begin{aligned} VI(\psi ^u, \psi ^v) = H(\psi ^u) + H(\psi ^v) - 2I(\psi ^u, \psi ^v). \end{aligned}$$

where $H(\psi )$ is the entropy of partition $\psi$ which divides *n* elements into *K* subsets, with the $k^{th}$ subset having $n_k$ elements, and is given by [2]:

2 $$\begin{aligned} H(\psi ) = - \sum ^{K}_{k=1} p(k) \log p(k), \end{aligned}$$

where *p*(*k*) is the probability of subset or cluster *k*, which is given in this approach by the cardinality of the subset as a proportion of the entire set: $p(k)=\frac{n_k}{n}$. $I(\psi ^u, \psi ^v)$, on the other hand, is the mutual information of partitions $\psi ^u$ and $\psi ^v$, and is given by:

3 $$\begin{aligned} I(\psi ^u, \psi ^v) = \sum _{k_i=1}^{K_i} \sum _{k_j=1}^{K_j} p(k_i, k_j) \log \frac{p(k_i, k_j)}{p(k_i)p(k_j)}, \end{aligned}$$

where $p(k_i, k_j)$ is the joint probability of subset $\psi ^u_{k_i}$ from partition $\psi ^u$ and subset $\psi ^v_{k_j}$ from partition $\psi ^v$. This is given by the proportion of the size of the intersection of subsets to the entire dataset: $p(k_i, k_j) = \frac{|\psi ^u_i \cap \psi ^v_j|}{n}$.

### Usefulness as a species delimitation partition metric

The information theoretic-based distances provided by Piikun are very flexible. For example, different organizations of different populations into sets of species may be analyzed together from multiple different inferences or publications, even if the numbers of individuals, populations, or genes sampled across these sources vary. Furthermore, there are no constraints on the relationships between the partitions considered, such as being nested or otherwise. Detailed discussion of the statistical characteristics and properties of this statistic are given in [2]. Here we present a conceptual description of the properties of this metric that make it useful for analysis of differences between species delimitation models.

A true metric or distance function, such as the variation of information, has the properties of non-negativity, symmetry, and the triangle equality [11]. These properties are useful in aligning with human intuition when interpreting values, as well as preferable in statistical or computational terms due to benefits in algorithm and data structure design or scaling up comparisons [2].

In addition to being a true metric, the variation of information is aligned with the lattice of the set of partitions. Formally, a partition lattice is an ordered set of partitions, where the order is defined by the refinement of the partitions. We can represent the set of all partitions of a particular set of elements as a *partition lattice* using the refinement order (a partition *U* is defined as a refinement of a partition *V* if every block in *U* is a subset of a block in *V*: we say that *U* is finer than *V*, or, equivalently, *U* is coarser than *V*). Conceptually, a partition lattice provides geometrical representation of all possible divisions of a set of elements, from the most granular, where each element forms its own subset, to the most general, where all elements belong to a single subset. This allows for the understanding of relationships between different partitions, offering insights into how small changes in groupings can lead to new partitions, and how these partitions are (or are not) nested within each other. A metric on partitions that is aligned along the lattice of partition sets has numerous advantages for interpretation of the disagreements between different partitions as well as for computation as neighbors in the metric space correspond to refinements in the partition lattice [36]. Such a metric takes into account the hierarchical nature of partitions, recognizing that partitions related through a series of refinements are closer to each other than partitions that differ more fundamentally. That is, under this metric, the nearest neighbor of a species delimitation partition is always obtained by “lumping” the two smallest species blocks of that partition, or vice versa via “splitting”, and never “moving” species from one block to another. This alignment with the partition lattice is not only useful for intuition, but may also have potential to provide benefits in designing inference or stochastic sampling algorithms (e.g., MCMC moves in partition space may be tuned or auto-adapted to finer vs. coarser movements depending on the quality of the parameter space). Third, the variation of information is convexely additive. This means that if two disjoint sets of species are submitted to a set of delineation algorithms, when we consider the union of the two sets of species, together with the algorithms results, the distances between partitions on the union are obtained by combining the distances on each species set proportionally to the set size. This property is very rare for a partition comparison criteria. In [36] it was proved that no other comparison criterion has all these intuitive properties.

Another useful property of this metric is *n*-invariance, i.e., the distance between two partitions is independent of the absolute number of elements [2]. This allows comparisons as well as combinations of distances of partitions of data sets of different sizes. For example, two independent studies of the system may report distances between species delimitation models, with the first study organizing dozens of lineages into species units while the other may involve hundreds or even greater. Despite this disparity in the number of elements being organized into higher-level groups or clusters, the distances between these models can be compared, as the critical quantity in is the relative proportion of the entire respective sets represented by the nominal species units in each partition [2]. Consider $X_1, X_2, X_3, \ldots$ etc. datasets, each consisting of some arbitrary number of samples of an arbitrary subset of populations of system *S*. Let $f_1, f_2, f_3, \ldots$ each map to the results of a particular inference algorithms or approach, expressed in the form of a particular clustering of the elements of the dataset, as optimized according the inferential model criteria. So each $f_i(X_j)$ produces a partition $P_{ij}$ of the elements in $X_j$.

The VI can, of course, compare partitions of the same dataset under different algorithms with no constraint, $VI(P_{ij} | P_{uv})$, is a valid distance for all *i*, *u*. While the VI cannot directly compare partitions between datasets of different sizes, i.e., $VI(P_{ij} | P_{uv})$ is invalid if $j \ne v$, and, indeed, no partition metric can, the *n*-invariance property of the VI allows *distances* between particular partitions to be compared across datasets, and this is something that no other partition metric can do. That is, for example, $VI(P_{ij} | P_{uj})$ can uniquely be summarized, compared, averaged, etc. across $j \in 1,2,3\ldots$, corresponding to datasets $X_1, X_2, X_3,\ldots$, to develop evaluations of a “marginalized” distance between inference approaches or algorithms.

The upper bound of this metric is given by $2 \log K$, where *K* is the maximum number of subsets in a partition, or by *log*(*n*), with *n* the number of elements (populations or individuals, depending on the approach), if any partition is possible [2]. Some researchers introduced a version of variation of information normalized to the range [0, 1] [35], which retains the metric properties of the variation of information. Piikun provides for the calculation of the normalized variation of information too. Unfortunately, while it is straight-forward enough to normalize by these known upper bounds, this will preclude comparison of distances across different datasets (in particular, datasets of different sizes), and it will remove the very intuitive additivity property of VI.

## Conceptual example

**Fig. 1:**
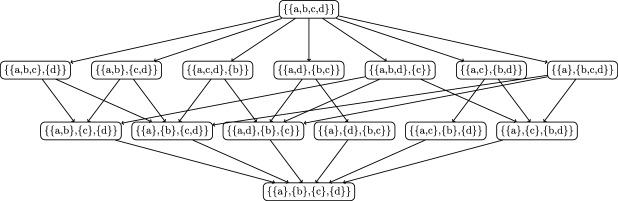
Partition lattice for a set of four elements, *a*, *b*, *c*, *d*. This Hasse diagram shows all possible ways of clustering elements into subsets, organized by refinement ordering. In a partition lattice, each node represents a distinct partition of the set, with edges directed from coarser partitions (fewer subsets) to finer partitions (more subsets). The coarsest partition at the top groups all elements together as *a*, *b*, *c*, *d*, while the finest partition at the bottom separates each element individually as *a*, *b*, *c*, *d*. The VI distance is aligned along this lattice, with the lowet VI distances corresponding to the smallest refinement

Figure 1 shows the partition lattice for a set of 4 elements, $\{a,b,c,d\}$, in the form of a Hasse diagram, which shows all possible ways to group these elements into subsets. Each node represents a distinct partition, with edges connecting coarser partitions (fewer subsets) to finer ones, with coarser partitions at the top and finer ones at the bottom, so moving downward indicates increasing refinement of the partition structure.

The VI distance between the partition consisting of a single set, $\{a,b,c,d\}$, and a partition that divides the set into two equal-sized subsets, $\{a,b\}$ and $\{c, d\}$, is $VI(\{abcd\} \mid \{ab\},\{cd\}) = 1$. In other cases, such as

$$\begin{aligned} VI(\{a\},\{b\},\{cd\} \mid \{ab\},\{cd\})&= VI(\{ab\},\{c\},\{d\} \mid \{ab,cd\}) \\&= VI(\{a\},\{b\},\{c\},\{d\} \mid \{a\},\{b\},\{cd\}) \\&= \frac{1}{2}, \end{aligned}$$

we are splitting half the data into two equal clusters while leaving the other half unchanged. Note that this value is the same regardless of how finely the remaining data is clustered, demonstrating convex additivity.

Moreover,

$$\begin{aligned} VI(\{abcd\} \mid \{a\},\{b\},\{c\},\{d\}) = 1 + \frac{1}{2} + \frac{1}{2} = 2, \end{aligned}$$

which results from alignment with the lattice of partitions.

The “match ratio” (MR) of [9] (originally implemented in LIMES, but also now in Piikun) is a similarity index in [0, 1], where 1 represents maximum similarity, and 0 represents complete dissimilarity.

For example:

$$\begin{aligned} MR(\{abcd\} \mid \{ab\},\{cd\}) = MR(\{abcd\} \mid \{a\},\{bcd\}) = MR(\{abcd\} \mid \{b\},\{acd\}) = 0, \end{aligned}$$

indicating complete dissimilarity.

Suppose we have *n* elements. Then

$$\begin{aligned} MR(\{a_1,a_2,\ldots , a_n\} \mid \{a_1\},\{a_2,\ldots , a_n\}) = 0, \end{aligned}$$

even though intuitively, splitting off a single point should be a negligible change for large *n*. In contrast,

$$\begin{aligned} VI(\{a_1,a_2,\ldots , a_n\} \mid \{a_1\},\{a_2,\ldots , a_n\}) \end{aligned}$$

approaches 0 as *n* grows.^2^

For further comparison:

$$\begin{aligned} VI(\{ab\},\{cd\} \mid \{a\},\{b\},\{c\},\{d\}) = \frac{1}{2} + \frac{1}{2} = 1, \end{aligned}$$

while

$$\begin{aligned} MR(\{ab\},\{cd\} \mid \{a\},\{b\},\{cd\}) = \frac{2}{5} = 0.4, \\ MR(\{a\},\{b\},\{cd\} \mid \{a\},\{b\},\{c\},\{d\}) = \frac{4}{7} \approx 0.57, \end{aligned}$$

and

$$\begin{aligned} MR(\{ab\},\{cd\} \mid \{a\},\{b\},\{c\},\{d\}) = 0. \end{aligned}$$

## Results: example exploratory discovery analysis

**Fig. 2:**
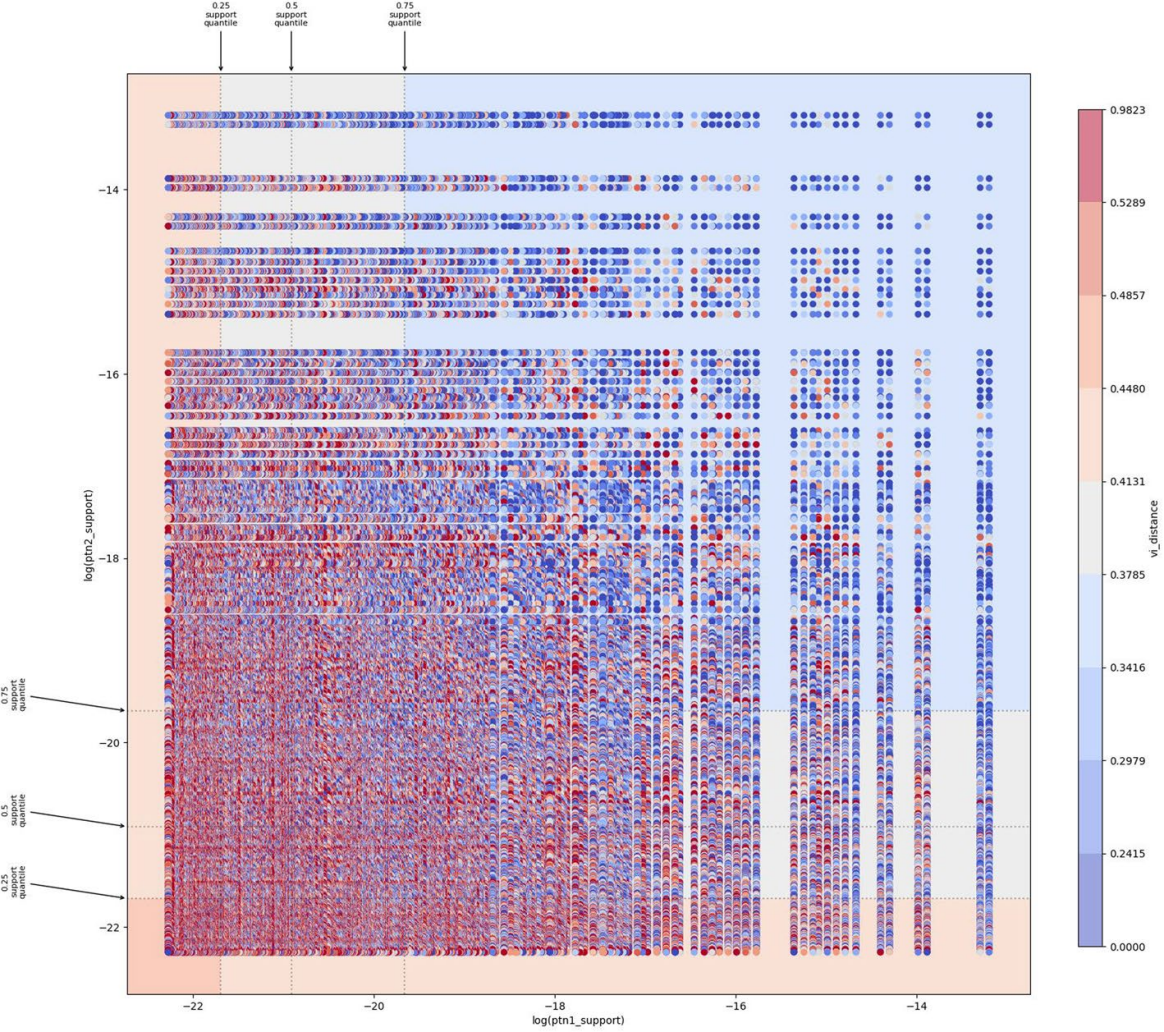
Understanding how the pairwise similarity of competing species delimitation models varies with their probability (support). The log(ptn_1) and log(ptn_1) axes represent species delimitation models (partitions) ordered by their log-scaled probabilities. Each point represents a pair of models; its color encodes the variation of information distances between them. The color gradient goes from blue to red, with blue indicating smaller distances while red indicating greater distances. The plot background is regionalized into 0.25, 0.5, and 0.75 quantiles based on support, with mean distance for partitions compared within each region indicated with the same color scheme. Note how partitions with high probabilities (upper-right area) are mutually close (VI-distance almost 0), while partitions with lower probabilities (lower-left area) are much more different (VI-distance over 0.91)

Here we focus on one of the visualizations offered by Piikun which gives us insight into how the “disagreement” between two partitions, as measured by their distance, might vary with respect to some value, trait, or attribute of each of the respective partitions. The visualization in Figure 2 shows how different species delimitation partition distances might vary in relation to the support (probability) of each partition in the comparison. Piikun was run on the 1000 most probable species delimitation models from a DELINEATE inference on a dataset of *Lionepha* beetles [37, available on the DELINEATE website]. The partitions with high probabilities (i.e., the upper-right area of Fig. 1) are mutually close. In contrast, the less probable partitions are much more dissimilar, pairwise (i.e., the lower-left area of Fig. 1), while remaining relatively similar to the group of probable partitions (i.e., the right edge of Fig. 1), and particularly to the most probable one. Hence, we know that there is essentially one good delimitation model (with small variations) for these data. We also see that even small changes in the model entrain significant changes in the support (see the gaps between the top-ranked models). Moreover, the pairwise similarities strongly suggest that the compact “core” formed of the most probable partitions must be the center of a “halo” of diverse, less probable partitions. This interpretation is made possible by the fact that the VI is a metric.

Further analysis (e.g., explicit enumeration and comparison of the subsets of the partitions) could then be used confirm that this is indeed because the species delimitation partitions with higher probabilities are disagreeing on the finer-scale splitting of species, while the less probable models, conflict with each other in more fundamental ways. Other datasets might indicate other relationships, e.g. where more supported models show more fundamental differences from one another, serving as the motivation or bases for more directed statistical analysis at the identified regions of partition space of interest.

## Conclusions

Piikun is a Python package of command-line tools for generating insight about *differences* between species delimitation models, in addition to evaluating them in terms of quality of inference or data. Piikun provides a general implementation of the variation of information criterion [2], a metric function returning distances between partitions of a set, that, in addition to being a metric has a range of particular characteristics that are especially useful when used to establish a metric space for species delimitation models, such as alignment along the lattice of partitions, which supports intuitive interpretation of the differences with reference to nesting of the models. As with all information theoretic approaches, the metric extracts signal from patterns in the data without reference to any mechanistic or phenomenological processes or assumptions, which allows species delimitation models to be compared across inferrential models and datasets. This combination of being a true metric, alignment along the lattice of partitions, and being able to compare not just species delimitation models, but essentially any partitioning of elements as long as the concepts can be represented as an arbitrary nesting of arbitrary labels expressible as a JSON list (with more specialized formats for associationg metadata), allows for the application of Piikun to provide insight into differences in domains well beyond systematics or even biology. With documentation that works the user through a complete workflow, a modular design for UNIX-pipeline style composibility, and the ability to be run directly without explicit userspace installation using ‘pipx‘ or easy local installation using ‘pip‘, Piikun will facilitate research in species development modeling as well as make a wider range of post-inferential analyses and visualization more accessible to empirical researchers in evolutionary biology and related fields. To understand the latter, note the impact and diversity of usage of true metric distances in the field of phylogenetics, such as the Robinson-Foulds (RF) metric [12], which is so critical for quantitative comparison of results across multiple studies, datasets, models, and inference frameworks that it is commonly considered a foundational element of the field [38]. We hope that the similar “general” (in terms of being inference model, theory, and data agnostic) metric space for species delimitation models that we provide here will also provide an analogous measure for the field of species delimitation analyses, adding to the ways that more insight can be generated not only from future studies but also by retrospective comparisons of or together with previous ones. 

## Availability and requirements

**Project name:** Piikun

**Project home page:** https://github.com/jeetsukumaran/piikun

**Operating system(s):** Platform independent

**Programming language:** Python

**Other requirements:** Python 3.10 or higher

**License:** New BSD License

**Any restrictions to use by non-academics:** None

## Data Availability

The results and analysis reported in the current study are available for download from: https://drive.google.com/drive/folders/1oN9yrsMrhIOdO-Y3aXAVW33d3RfVnbY9?usp=sharing, while the species delimitation models analyzed here are from a DELINEATE inference on a dataset of *Lionepha* beetles [available on the DELINEATE website]?.

## Footnotes

1 “sparrowhawk” in the Kumeyaay language [25]. San Diego State University is built on Kumeyaay land.

2 The exact expression is $\frac{1}{n}\log n + \frac{n-1}{n}\log \frac{n}{n-1}$.

## Declarations

### Code availability

The source code, documentation, example data sets, etc. are available for download, installation, or direct usage from the Piikun code repository: https://github.com/jeetsukumaran/piikun.
